# Emerging role of N-myc downstream-regulated gene 2 (NDRG2) in cancer

**DOI:** 10.18632/oncotarget.6228

**Published:** 2015-10-25

**Authors:** Wei Hu, Chongxi Fan, Peng Jiang, Zhiqiang Ma, Xiaolong Yan, Shouyin Di, Shuai Jiang, Tian Li, Yedong Cheng, Yang Yang

**Affiliations:** ^1^ Department of Biomedical Engineering, The Fourth Military Medical University, Xi'an, China; ^2^ Department of Thoracic Surgery, Tangdu Hospital, The Fourth Military Medical University, Xi'an, China; ^3^ Department of Orthopaedics, The 82th Hospital of PLA, Huaian, China; ^4^ Department of Aerospace Medicine, The Fourth Military Medical University, Xi'an, China

**Keywords:** N-myc downstream-regulated gene 2, cancer, pathological processes, molecular pathways

## Abstract

*N-myc downstream-regulated gene 2 (NDRG2)* is a tumor suppressor and cell stress-related gene. NDRG2 is associated with tumor incidence, progression, and metastasis. NDRG2 regulates tumor-associated genes and is regulated by multiple conditions, treatments, and protein/RNA entities, including hyperthermia, trichostatin A and 5-aza-2′-deoxycytidine, which are promising potential cancer therapeutics. In this review, we discuss the expression as well as the clinical and pathological significance of NDRG2 in cancer. The pathological processes and molecular pathways regulated by NDRG2 are also summarized. Moreover, mechanisms for increasing NDRG2 expression in tumors and the potential directions of future NDRG2 research are discussed. The information reviewed here should assist in experimental design and increase the potential of NDRG2 as a therapeutic target for cancer.

## INTRODUCTION

Cancer represents a large group of complex and multifactorial diseases that involve abnormal cell growth with the potential to invade other tissues [1]. Cancer accounted for approximately 8 million deaths in 2010, and invasive cancer was the leading cause of death in the developed world and the second leading cause of death in the developing world [2, 3]. Despite recent advances, effective clinical management remains elusive because of intra-tumoral heterogeneity and therapeutic resistance [4–6]. Therefore, it is essential to investigate the pathophysiology of cancer and identify novel therapies [7, 8]. Notably, associations between cancer (including lung cancer, prostate cancer, liver cancer, colorectal cancer and breast cancer) and N-myc downstream-regulated gene 2 (NDRG2) have been reported [2, 9–15]. Thus, NDRG2 may be a promising target for cancer.

The NDRG family consists of *NDRG1, NDRG2, NDRG3* and *NDRG4* [16, 17]. This family of proteins is characterized by an esterase/lipase/thioesterase active site serine and an α/β hydrolase fold of approximately 220 amino acids [16–19]. *NDRG2* is an important member of the NDRG family and is located at chromosome 14q11.2. The structure and tissue distribution of NDRG2 have been previously studied and reviewed [20]. *NDRG2* has been suggested to be a tumor suppressor and cell stress-related gene that is involved in cellular metabolic processes, such as hormone, ion, and fluid metabolism [21–23], and in stress responses, such as those to hypoxia and lipotoxicity [24, 25]. However, the associations between NDRG2 and cancer and the corresponding mechanistic details require intensive research.

This review focuses on the latest progress regarding the associations between NDRG2 and cancer. First, the expression as well as the clinical and pathological significance of NDRG2 in cancer is introduced. Then, we summarize how NDRG2 regulates pathological processes and molecular pathways in tumors and discuss mechanisms for increasing NDRG2 expression. Finally, potential directions for future NDRG2 research are discussed. The information compiled here comprehensively characterizes NDRG2 activity related to cancer, thus potentially aiding in the design of experimental research and promoting NDRG2 as a therapeutic target for cancer.

## NDRG2 AND THE NDRG FAMILY

The term “*NDRG*” was first used by Shimono et al. [26] for the *Ndr1* gene, which is up-regulated in *N-Myc*-knockout mouse embryos. The NDRG proteins (NDRG1, NDRG2, NDRG3 and NDRG4) are included within the α/β hydrolase group of enzymes, despite the lack of a hydrolytic catalytic site and a deficiency in enzyme function [16, 18, 27, 28]. Although the identity at the residue level is approximately 57-65% among members [17, 29], each NDRG family member forms a separate homology cluster across multiple species with specific and functionally divergent roles [16]. Phylogenetic analyses have revealed that NDRG1 and NDRG3 belong to one subfamily, whereas NDRG2 and NDRG4 belong to another subfamily [17]. The NDRG proteins are characterized by an esterase/lipase/thioesterase active site serine and an α/β hydrolase fold of approximately 220 amino acids [16–19]. The detailed structural features of human NDRG proteins have been discussed by Veerle et al. [16].

**Figure 1:**
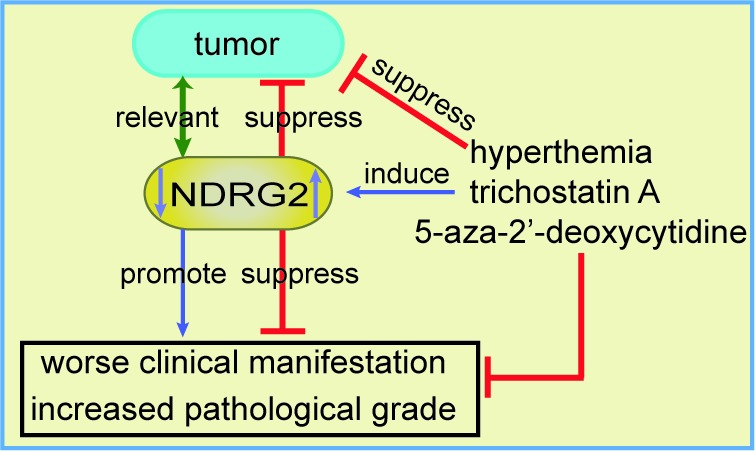
The association between NDRG2 and cancer and NDRG2 regulation in tumors The down-regulation of NDRG2 is associated with tumor incidence, although there is insufficient evidence for a causal relationship, and NDRG2 down-regulation is associated with worse clinical manifestations and increased pathological grade. Hyperthermia, trichostatin A and 5-aza-2′-deoxycytidine up-regulate the expression of NDRG2, which may further inhibit tumor development.

NDRG proteins have important roles in cell proliferation and differentiation. Notably, NDRG protein expression appears to positively correlate with progressive stages of differentiation. Low expression levels are detected at a relatively early embryonic stage, and expression levels are increased in postnatal and mature animals [16]. *NDRG3* and *NDRG4* respond to stress [32, 33] in addition to their roles as tumor-related genes [30, 31]. NDRG1 has been demonstrated to be negatively correlated with tumor progression [34–37]. Currently, there is no evidence demonstrating that NDRG1 acts as a transcription factor, and it lacks nuclear targeting sequence [38]. However, NDRG1 may affect other transcription factors, such as nuclear factor-kappa B (NF-κB), mothers against decapentaplegic homolog 4 (Smad4) and others [39–41]. The activation of NDRG1 and NDRG2 are activated in a similar manner related to phosphorylation [42–44]. NDRG2 has been shown to participate in ischemia-reperfusion injury [45], Alzheimer's disease [46, 47], depression [48, 49] and hypoxia [25, 50]. The role of NDRG2 in cancer has attracted increasing attention, and this topic will be discussed below (Figure 1 and Table 1).

**Table 1:** Role of NDRG2 in cancer

Tumor type	Expression level of NDRG2	Correlation between the NDRG2 expression level with		Regulators	Effect of over-expression of NDGR2	Reference No
		clinical significance	pathological significance			
Gastric cancer	mRNA and protein levels are lower	Survival rate of NDRG2-negative patients is lower. Silenced NDRG2 is associated with worse prognosis and shorter disease-free survival.	NDRG2 methylation and down-regulated NDRG2 are negatively related to depth of tumor invasion, Borrmann classification and TNM stage.	5-aza-2′-deoxycytidine and trichostatin A	Inhibit invasion	[60, 61, 71, 75]
				Hyperthermia	Increase apoptosis rate	
Colorectal cancer	mRNA and protein levels are lower	There is a trend for NDRG2 level to decrease with increasing Dukes' stage. Patients with reduced level of NDRG2 mRNA have a statistically significantly shorter disease-free survival and overall survival duration.	There is a trend for NDRG2 level to decrease with tumor invasion depth and histology grading.	--	--	[13, 14, 62–64, 68, 69]
ESCC	protein is lower	The expression of NDRG2 is inversely associated with clinical stage, patients' vital status and 5-year overall survival rate.	The expression of NDRG2 is inversely associated with TNM classification, and histological differentiation.	--	Reduce cell proliferation, colony formation and DNA replication activity	[58, 70]
Hepatocellular carcinoma	mRNA and protein levels are lower	NDRG2 down-regulation in patients is accompanied with elevated AFP serum level, portal vein thrombi, recurrence and lower survival rate.	NDRG2 down-regulation in patients with late TNM stage, infiltrative growth pattern, poor differentiation grade, nodal/distant metastasis and tumor invasion.	Non-steroidal FXR agonists	Inhibit tumor growth and metastasis potential of corresponding cells	[9–11, 59, 73, 74]
				Sh-NDRG2	Enhance EMT	
				Ad-NDRG2	Increase apoptosis rate	
Gallbladder carcinoma	--	Patients with NDRG2 negative expression correlate with worse prognosis and lower survival rate.	Down-regulation of NDRG2 tends to show deeper invasion depth and higher TNM stage.	--	--	[55]
Pancreatic cancer	mRNA is lower	There exists a significant association between poor prognosis and NDRG2-negative expression.	--	--	--	[10, 72]
Glioblastoma	protein is lower	Survival rate of patients is significantly negative with NDRG2 expression level.	Glioma tumor grade is significantly negative with NDRG2 expression level.	cDNA encoding hNDRG2	Reduce the cell proliferation	[19, 54–56]
Astrocytomas	mRNA and protein levels are lower	NDRG2 expression is positively correlated with the life span of astrocytoma patients.	NDRG2 expression is negatively correlated with pathological grading.	--	--	[51, 56]
MeningiomaA6:A6:B14	mRNA and protein levels are lower	--	--	--	--	[52]
Neuroblastoma		--	--	--	Inhibit cell proliferation	[57]
Renal cell carcinoma	mRNA and protein levels are lower	The down-regulation of NDRG2 expression is associated with higher tumor recurrence and lower survival rate.	The down-regulation of NDRG2 expression is negatively associated with TNM stage, tumor magnitude, nuclear grade, Fuhrman's grade and tumor invasion.	--	Inhibit tumor cell growth, migration and invasion	[79–82]
Prostate cancer	protein is lower	Low NDRG2 expression is significantly associated with short recurrence-free survival and overall survival.	The down-regulation of NDRG2 in prostate cancer tissues is significantly correlated with advanced pathological stage, positive metastatic status and high Gleason score.	Ad-NDRG2	Inhibit tumor growth and invasion	[77, 78]
Bladder cancer	protein is lower	--	The NDRG2 level is negatively correlated with tumor grade and pathologic stage.	LEN-NDRG2	Inhibit cell proliferation	[12]
Breast cancer	mRNA is lower	Patients with high NDRG2 expression have better disease-free survival and overall survival.	NDRG2 overexpression suppresses breast cancer cell adhesion and invasion.	--	--	[21, 83, 84]
Lung cancer	protein is lower	NDRG2 level is negatively correlated with UICC stage, and positively correlated with survival time.	NDRG2 level is negatively correlated with pathological metastasis and TNM stage.	--	--	[15, 86]
Thyroid cancer	mRNA and protein levels are lower	--	There is no significant correlation between NDRG2 expression and distant metastases.	--	--	[83, 87]
Fibrosarcoma	--	--	Tumor migration is significantly reduced by NDRG2.	Injected with NDRG2 cells	Inhibit cell proliferation	[88]
Oral squamous-cell carcinoma	mRNA is lower	--	Induction of NDRG2 expression significantly inhibits cell proliferation.	--	--	[89]
Myeloid leukemia	protein is lower	--	--	--	--	[90]

## ASSOCIATIONS BETWEEN NDRG2 AND CANCER

The associations between NDRG2 and cancer have been reported in neurologic tumors [19, 51–57], gastrointestinal tumors [9–11, 13, 14, 55, 58–75], genitourinary tumors [12, 76–82], breast cancer [21, 83–85], lung cancer [15, 86], thyroid cancer [83, 87], fibrosarcoma [88], oral squamous-cell carcinoma [89], myeloid leukemia [90] and cervical cancer (Hela cells) [25] (Table 1). Collectively, NDRG2 expression is associated with the clinical features of tumors. NDRG2 levels are positively correlated with tumor differentiation but negatively correlated with lymph node metastasis and TNM stage. Furthermore, NDRG2 levels tend to decrease with tumor invasive depth and increasing grade (Table 1 and Figure 1). Epigenetic silencing of the *NDRG2* promotor has been found in the majority of primary tumors, which may elicit resistance to anticancer drugs. However, whether NDRG2 down-regulation is a cause or a consequence of the progression from normal tissue to cancerous tissue remains unclear. NDRG2 down-regulation is associated with cancer development and progression, including such features as malignant clinical manifestations and increased pathological grade. NDRG2 is a relevant biomarker for predicting aggressive behavior, tumor recurrence and overall patient survival, independently or in combination with other factors, such as CD24, phospho-STAT3, and HOXD1. Ad-NDRG2, LEN-NDRG2, and B16F10-NDRG2 injections as well as other interventions that increase NDRG2 expression may control tumor progression. Therefore, NDRG2 up-regulation may be a promising therapeutic strategy for the treatment of cancer.

## MOLECULAR TARGETS OF NDRG2

To understand the role of NDRG2 in cancer and provide insight into its mechanisms of action and potential applications, we have focused on the molecular basis of NDRG2 activity in this section. As a master switch for cell proliferation and differentiation, NDRG2 mainly exerts biological activity by modulating protein expression and phosphorylation.

### Proliferation-associated proteins and pathways

Cyclin D1 belongs to the highly conserved cyclin family, whose members are characterized by a dramatic periodicity in protein abundance during the cell cycle [91]. The overexpression of cyclin D1 alters cell cycle progression, which may contribute to tumorigenesis; indeed, cyclin D1 overexpression has been observed in various cancers [91]. In SW620 colon carcinoma cells, the induction of NDRG2 decreases c-Jun phosphorylation at Ser63, which is followed by the attenuation of the transcriptional activator AP-1 (activator protein-1). This further down-regulates cyclin D1 and results in cell cycle arrest at G1/S [67]. In addition, NDRG2 siRNA can reverse the phenotype of NDRG2-expressing cells, recovering c-Jun phosphorylation and cyclin D1 expression as well as cell proliferation [67]. In conclusion, NDRG2 modulates intracellular signals to inhibit cell proliferation by suppressing c-Jun phosphorylation and cyclin D1 expression.

P38 mitogen-activated protein kinase (MAPK) plays an important role in key cellular processes related to cancer [92, 93]. Liu et al. [94] conducted a microarray study to determine the expression profile of NDRG2-overexpressing HepG2 cells and found that p38 phosphorylation was increased by NDRG2. Furthermore, in malignant breast cancer cells, NDRG2 overexpression specifically inhibits suppressor of cytokine signaling 1 (SOCS1) phosphorylation and induces the phosphorylation of p38 MAPK [95]. Inhibiting p38 MAPK activity blocks the induction of SOCS1 expression by NDRG2 [95]. Therefore, NDRG2 expression can increase the phosphorylation of p38 MAPK, which further inhibits the phosphorylation of SOCS1 and suppresses tumor proliferation. Interestingly, inhibitors of p38 MAPK have attracted attention in research related to cancer treatment [92, 93]; however, NDRG2 exerts anti-tumor effects via the activation of p38 MAPK.

### Migration/metastasis/invasion-associated proteins and pathways

β-catenin is a dual-function protein that regulates cell-cell adhesion and gene transcription [96]. Mutation and overexpression of β-catenin are associated with the incidence of cancer. NDRG2 inhibits *c-Myc* expression by suppressing the expression of β-catenin [97], and the possible mechanisms for this effect have been investigated. The nuclear localization of β-catenin and the inappropriate activation of T-cell factor (TCF)/lymphoid enhancer factor (LEF)-mediated transcription appear to be important processes for establishing and maintaining cancer stem cells [98]. The introduction of wild-type, but not mutant, *NDRG2* reduces the transcriptional activity of TCF/LEF [68]. Intracellular β-catenin levels are reduced in *NDRG2*-transfected SW620 cells, and the suppression of β-catenin stability and TCF/LEF activity is mediated through the activation of glycogen synthase kinase 3β (GSK-3β) by NDRG2. The attenuation of TCF/β-catenin signaling by NDRG2 contributes to the maintenance of healthy tissues and the suppression of tumor metastasis [68].

E-cadherin, a classical member of the cadherin superfamily, is a well-known tumor suppressor [99]. Positive correlations between the expression of E-cadherin and NDRG2 have been observed in cancer [66, 79]. Snail is a zinc-finger transcriptional repressor that has been shown to mediate the regulation of E-cadherin expression by NDRG2 [66]. The enhancement of GSK-3β activity by NDRG2 overexpression causes the proteasomal degradation of Snail followed by the transcriptional de-repression of E-cadherin. In renal cell carcinoma and colon cancer cells, NDRG2 can recover E-cadherin expression, and this effect can be reversed by NDRG2 siRNA [66, 79]. Through GSK-3β activation, NDRG2 promotes cell density-regulated E-cadherin expression and exerts anti-tumor effects.

Transforming growth factor beta 1 (TGFβ1), a member of the multifunctional set of TGFβ peptides, controls cell proliferation and differentiation [100]. Down-regulation of the TGFβ pathway is associated with cancer development and progression. Furthermore, dysregulation of TGFβ activation and signaling can result in apoptosis [101]. NDRG2 antagonizes TGFβ1-mediated tumor cell invasion by down-regulating the expression of matrix metalloproteinase 2 (MMP2) and laminin 332 pathways, with concomitant suppression of Rho GTPase activity [11].

Proteins in the MMP family are involved in the breakdown of the extracellular matrix in normal physiological processes, such as embryonic development, reproduction, and tissue remodeling, as well as in disease processes, such as arthritis and metastasis [102]. There is evidence for an association between MMPs and cancer [103, 104]. NDRG2 overexpression inhibits the expression of MMP2 and MMP9 in clear cell renal cell carcinoma (CCRCC) and hepatocellular carcinoma (HCC) [11, 65, 81]. Moreover, *NDRG2* knockdown increases cell invasion, which is rescued by treating HepG2 cells with the extracellular signal-regulated kinase (ERK) inhibitor PD98059, thus revealing that ERK1/2 phosphorylation is reduced in NDRG2-overexpressing cells and can further increase MMP expression [65]. In HCC cells, phospho-ERK1/2 levels were significantly decreased when NDRG2 was overexpressed [74]. There are several mechanisms by which NDRG2 suppresses MMP expression. In fibrosarcoma and murine melanoma, NDRG2 expression significantly suppresses tumor invasion by inhibiting MMP activity, which is regulated by NF-κB signaling [88]. The suppression of MMP2 can be reversed by the activation of TGFβ1 in response to NDRG2 overexpression [11]. MMPs, especially MMP9, can also be suppressed via the activation of bone morphogenetic protein-4 (BMP-4) [105], which will be discussed further in the next section. Thus, NDRG2 overexpression can suppress MMPs via various mechanisms, which further suppress tumor invasion.

BMP-4 is a member of the BMP family that stimulates tissue formation and differentiation, and the abnormal expression of BMP-4 may be associated with tumor development [106]. In breast cancer cells, the specific induction of active BMP-4 is exclusively observed in breast cancer cells expressing *NDRG2* but not in control breast cancer cells, and NDRG2 expression inhibits the mRNA expression of several MMPs and the gelatinolytic activity of MMP9 [105]. Neutralization of BMP-4 in *NDRG2*-expressing breast cancer cells results in the rescue of MMP9 mRNA expression and migration capacity. Additionally, treatment with recombinant BMP-4 dramatically suppresses MMP9 mRNA expression and gelatinolytic MMP9 activity as well as the migration and invasion of MDA-MB-231 cells and PMA-treated MCF-7 cells [105]. Thus, the induction of BMP-4 by NDRG2 inhibits the metastatic potential of cancer cells, specifically by suppressing MMP9 activity.

CD24 mediates cell-cell interactions as a surface marker that is expressed on cancer cells [107]. Jaggupilli et al. [107] analyzed the significance of CD24 as a cancer stem cell surface marker. There is a negative correlation between NDRG2 expression and CD24 expression in gallbladder carcinoma (GBC), HCC, breast cancer and lung adenocarcinoma [55, 73, 84]. NDRG2 inhibits CD24 expression and further suppresses tumor adhesion, migration and invasion in HCC [73]. CD24 may be a downstream target of NDRG2 in cancer, and the combination of CD24 and NDRG2 is considered an effective biomarker of tumor behavior.

The phosphatidylinositol 3-kinase (PI3K)/protein kinase B (Akt) pathway plays a critical role in malignant transformation as well as in tumor growth and metastasis [108, 109]. The majority of oral squamous cell carcinoma (OSCC) cell lines have activated PI3K/Akt signaling. Furthermore, positive p-Akt staining is inversely correlated with decreased NDRG2 expression in OSCC samples with moderate to poor differentiation [89]. Moreover, the enforced expression of NDRG2 in HSC-3 cells decreases the phosphorylation of Akt at Serine 473 [89]. In malignant breast cancer, NDRG2 overexpression has been demonstrated to specifically inhibit Akt phosphorylation [95]. Thus, NDRG2 contributes to the genesis and progression of OSCC and breast cancer partly through the inhibition of PI3K/Akt signaling.

### Survival-associated proteins and pathways

Stress-activated protein kinase/c-Jun NH(2)-terminal kinase (SAPK/JNK) activation occurs in response to cellular stresses and extracellular signals. The activation of SAPK/JNK plays a key role in regulating cell survival, apoptosis, and proliferation [110]. NDRG2 overexpression in malignant breast cancer cells specifically induces the phosphorylation of SAPK and JNK, which contributes to the survival of normal cells and the apoptosis of tumor cells [95].

Bcl-2-associated X protein (Bax) is a member of the Bcl-2 gene family that functions as an apoptotic activator [111]. Bax has been demonstrated to be deregulated through mutation or the inhibition of expression, which increases resistance to chemotherapy and radiotherapy [111]. However, in Hela cells, NDRG2 has been shown to abolish the radiation-induced up-regulation of Bax, to contribute to the survival of Hela cells, and to prevent tumorigenesis [25].

### Energy metabolism-associated proteins and pathways

Glucose transporter 1 (GLUT1), also known as solute carrier family 2, is a uniporter protein that is encoded by the *SLC2A1* gene in humans. GLUT1 facilitates the transport of glucose across the plasma membrane of mammalian cells, and GLUT1 overexpression is a prognostic indicator for cancer [112]. NDRG2 expression is negatively correlated with GLUT1 expression in breast carcinoma tissues; NDRG2 promotes GLUT1 protein degradation but does not affect GLUT1 transcription [21]. In colorectal cancer cells, NDRG2 suppresses the expression of GLUT1 as well as of other glucose transporters and catalytic enzymes involved in glycolysis and glutaminolysis, including hexokinase 2 (HK2), pyruvate kinase M2 isoform (PKM2), lactate dehydrogenase A (LDHA), the glutamine transporter ASC amino acid transporter 2 (ASCT2) and glutaminase 1 (GLS1) [97].

### Transcription factors and genes

Signal transducer and activator of transcription (STAT) activation within tumor cells contributes to pro-survival phenotypes [113, 114]. Signal transducer and activator of transcription 3 (STAT3) plays important roles in tumor cell proliferation, survival, invasion and immunosuppression [115, 116]. In addition to its established role as a transcription factor in cancer, STAT3 regulates mitochondrion function and gene expression through epigenetic mechanisms [115]. Moreover, STAT3 activation in both resting and IGF-stimulated cells is remarkably inhibited by NDRG2 expression [95]. The recovery of STAT3 phosphorylation can further block the inhibition of SOCS1 expression by NDRG2 [95]. When NDRG2 was overexpressed in HCC cells, STAT3 phosphorylation levels were significantly decreased [74]. These data demonstrate that NDRG2 expression inhibits STAT3 activation, thus affecting the expression of several genes and contributing to anti-tumor effects.

NF-κB constitutes a family of transcription factors involved in the regulation of a wide variety of biological responses, including cytokine production and cell survival. NF-κB regulates the expression of genes involved in many processes that play key roles in the development and progression of cancer, such as cell proliferation, migration and apoptosis. Aberrant or constitutive NF-κB activation has been observed in cancer [117, 118]. Kim et al. [88] found that NDRG2 suppresses NF-κB activity and affects cancer cell invasion by suppressing MMPs in colon carcinoma cells.

The *Cockayne syndrome group B protein (ERCC6)* gene is an NDRG2-inducible target gene in HCC [119]. *ERCC6* gene expression is suppressed by Ad-NDRG2 in combination with rAd-p53, but the NDRG2-enhanced apoptosis is reversed after transfection with *ERCC6* [57]. *Cysteine-rich protein 61 (CYR61)* is an important proliferation-related gene that can be inhibited by NDRG2 overexpression [59]. NDRG2 expression also inhibits the expression of epithelial-to-mesenchymal transition (EMT)-related genes, such as *Snail, Slug,* and *Smad-interacting protein 1 (SIP1)*, and decreases EMT signaling in renal cell carcinoma [79].

Overall, NDRG2 regulates several transcription factors, which further impact the expression of downstream genes. NDRG2 has been reported to modulate tumor-related genes; however, whether NDRG2 is a transcription factor itself or acts via other transcription factors is unclear.

**Figure 2:**
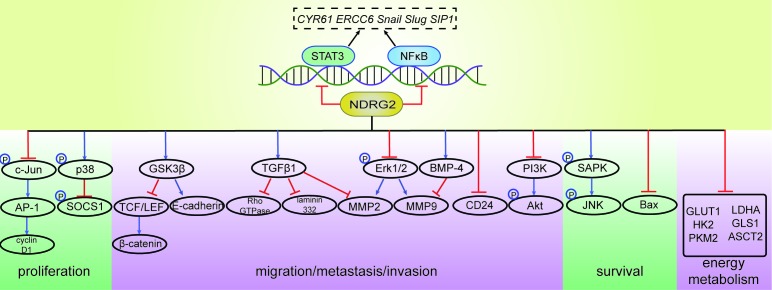
Molecular targets of NDRG2 NDRG2 acts on various proteins to inhibit tumor proliferation; suppress migration, metastasis and invasion; maintain normal cell survival; and interrupt energy metabolism in tumors. Several genes are regulated by NDRG2, which may function via interactions with transcription factors, such as NF-κB and STAT3, which are suppressed by NDRG2. The evidence supporting NDRG2 as a transcription factor itself is currently limited. NF-κB, nuclear factor-kappa B; STAT3, signal transducer and activator of transcription 3; *CYR61*, *Cysteine-rich protein 61*; *ERCC6*, *Cockayne syndrome group B protein*; *SIP1*, *Smad interacting protein 1*; AP-1, activator protein-1; SOCS1, suppressor of cytokine signaling 1; GSK-3β, glycogen synthase kinase 3β; TCF, T-cell factor; LEF, lymphoid enhancer factor; TGFβ1, transforming growth factor beta 1; Erk, extracellular signal regulated kinase; MMP, proteins of the matrix metalloproteinase; BMP-4, bone morphogenetic protein-4; PI3K, phosphatidylinositol 3-kinase; Akt, protein kinase B; SAPK, stress-activated protein kinase; NH(2)-terminal kinase, c-Jun; Bax, Bcl-2-associated X protein; GLUT1, glucose transporter 1; HK2, hexokinase 2; PKM2, pyruvate kinase M2 isoform; LDHA, lactate dehydrogenase A; ASCT2, ASC amino acid transporter 2; GLS1, glutaminase 1.

Thus, NDRG2 exerts anti-tumor effects through various mechanisms that are summarized in Figure 2.

## NDRG2-TARGETED PROCESSES

### Promotion of apoptosis

Apoptosis is the process of programmed cell death that occurs in multicellular organisms and is characterized by blebbing, cell shrinkage, nuclear fragmentation, chromatin condensation, and chromosomal DNA fragmentation [120]. The anti-tumor effects of NDRG2 have been demonstrated to be closely related to the promotion of apoptosis. Substantially more apoptotic cells are observed in NDRG2-expressing CCRCC cells than in control cells [82]. In the ESCC cell lines EC9706 and EC109, NDRG2 overexpression markedly promotes apoptosis [70]. The phosphorylation of NDRG2 can be increased by hyperthermia (HT), which further promotes the apoptosis of MKN28 cells [71]. Cao et al. [59] found that Ad-NDRG2 enhances the p53-mediated apoptosis of HCC cells by attenuating nucleotide excision repair. NDRG2 has also been shown to protect Hela cells from radiation-induced apoptosis [25]. Collectively, NDRG2 promotes tumor cell apoptosis but inhibits the apoptosis of normal cells.

### Resetting cell proliferation

One characteristic of a tumor is uncontrolled cell proliferation. Resetting of the cell cycle, including the G1, S, G2 and M phases, can be observed during tumorigenesis. A Gene Ontology biological process analysis revealed that NDRG2 overexpression elicits the up-regulation of genes related to the G protein signaling pathway and the down-regulation of gene sets related to the M phase of the cell cycle, which is consistent with cell cycle analyses [94]. A signaling pathway analysis demonstrated reduced glycosylphosphatidylinositol (GPI)-anchor biosynthesis and protein degradation [94]. Ma et al. [82] also found that NDRG2 expression can induce G1 arrest. Cell cycle arrest at G1/S was also observed after the introduction of NDRG2 into SW620 cells [67]. The resetting of the cell cycle in tumors can be effectively inhibited by NDRG2 expression.

### Inhibition of angiogenesis

Intra- and peri-tumoral angiogenesis is critical for tumor growth and metastasis. Kim et al. [88] found that angiogenesis is clearly observed in tumors after injection with B16F10-mock cells, whereas angiogenesis is impaired in tumors after injection with *NDRG2*-expressing murine melanoma cells. Thus, the inhibition of angiogenesis may contribute to the anti-tumor effects of NDRG2.

### Suppression of energy metabolism

The glucose transporter GLUT1 catalyzes the facilitative diffusion of glucose into erythrocytes and is responsible for glucose supply to the brain and other organs [112]. NDRG2 suppresses the expression of transporters and catalytic enzymes, which provide bioenergy and biomaterials for cancer cell proliferation and tumor progression, thereby playing an important role in inhibiting glycolysis and glutaminolysis and restraining tumor cell metabolism [21, 97].

**Figure 3:**
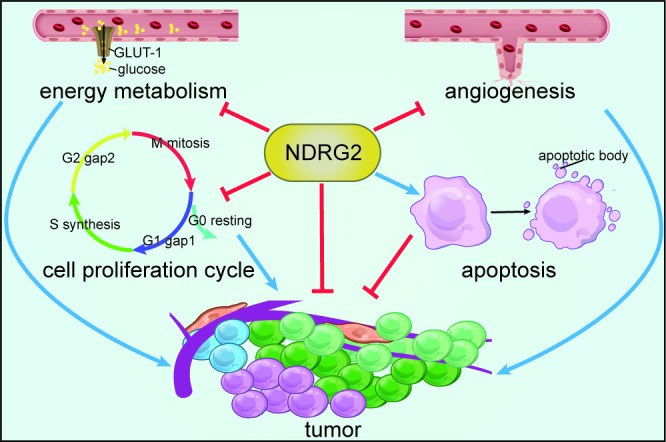
Processes targeted by NDRG2 NDRG2 exerts anti-tumor effects by promoting apoptosis, arresting cell proliferation, inhibiting angiogenesis and suppressing energy metabolism.

Collectively, tumors exhibit numerous abnormal behaviors, including aberrant proliferation, angiogenesis and metabolism. NDRG2 exerts anti-tumor effects by regulating various processes, including promoting apoptosis, arresting cell proliferation by influencing cell cycle, inhibiting angiogenesis and suppressing energy metabolism, which may provide attractive strategies for therapeutic interventions in human cancer (Figure 3).

## REGULATION OF NDRG2 EXPRESSION IN CANCER

As a N-Myc downstream-regulated gene, NDRG2 expression can be suppressed by N-Myc [16]. In this section, we focus on the regulation of NDRG2 expression in cancer (Table 2).

**Table 2:** Regulation of NDRG2 expression in cancer

Factors	Tumor	Effect on NDRG2	Reference No
DNA methylation	glioma, hepatocellular carcinoma, meningioma and gastric carcinoma	represses the expression of NDRG2	[11, 52, 60, 64, 75, 121]
DNA histone deacetylase	pancreatic cancer cells	represses the expression of NDRG2	[72]
p53	clear cell renal cell carcinoma cells	upregulates the expression of NDRG2	[82]
HIF-1	Hela cells	upregulates the expression of NDRG2	[25]
FXR	hepatoma cells	upregulates the expression of NDRG2	[9]
Akt	gastric cancer cells	induces the phosphorylation of NDRG2	[71]
Dp44mT	hepatocellular carcinoma cells	upregulates the expression of NDRG2	[74]
miR-650	colorectal cancer cells	represses the expression of NDRG2	[64]

Mutational analyses of the entire NDRG2 coding sequence have not revealed tumor-associated DNA sequence alterations [10, 56]. However, epigenetic alterations play a key role in tumorigenesis. The methylation rate of the NDRG2 promoter region is significantly higher in glioma, HCC, meningioma, and gastric carcinoma tissues compared with adjacent normal tissues, which may down-regulate NDRG2 [11, 52, 60, 64, 75, 121]. However, hyper-methylation was not detected in either pancreatic cancer cell lines or surgically resected specimens [72], whereas a histone deacetylase inhibitor up-regulates NDRG2 expression in pancreatic cancer cell lines that express low levels of NDRG2 [72]. These results demonstrate that altered NDRG2 expression levels during tumorigenesis are caused by epigenetic alterations such as increased methylation or histone deacetylase activity, not mutations in the coding region of NDRG2.

NDRG2 expression can be regulated by several factors. Ma et al. [82] found that p53 up-regulates NDRG2 expression in CCRCC. Hypoxia inducible factor 1 (HIF-1) is the key mediator of hypoxia signaling pathways and is involved in hypoxia-induced radioresistance [25]. NDRG2 is a target gene of HIF-1 and is up-regulated by hypoxia and radiation in an HIF-1-dependent manner, which decreases the sensitivity of Hela cells to radiation [25]. Farnesoid X receptor (FXR) directly increases NDRG2 transcription via IR1-type element(s) in the first introns of the human, mouse and rat *NDRG2* genes [9]. NDRG2 mRNA can be induced by non-steroidal FXR agonists in the mouse liver and by the ectopic expression of human FXR [9]. Dp44mT, an iron chelator, up-regulates NDRG2, ultimately suppressing EMT and inhibiting metastasis in HCC [74]. NDRG2 phosphorylation affects NDRG2 protein activity and is induced by HT in an Akt-dependent manner in gastric cancer cells [71].

The transcriptional regulation by endogenous small noncoding RNA, including microRNA, may be a potential method for regulating gene expression in human cancer. MicroRNA-650 (miR-650) targets a homologous DNA region in the promoter region of the *NDRG2* gene and represses its expression [64]. A reporter assay with the 3′ untranslated region of *NDRG2* cloned downstream of the luciferase gene showed reduced luciferase activity in the presence of miR-650, indicating that miR-650 is a direct inhibitor of NDRG2 expression [64].

## POTENTIAL FUTURE DIRECTIONS

The associations between NDRG2 expression and tumor incidence as well as clinical and pathological tumor behavior have been clarified. However, whether NDRG2 down-regulation is a cause or a consequence of the progression from normal tissue to carcinoma must be addressed.

*NDRG2* has been identified as a specific tumor suppressor gene. However, whether NDRG2 can be used as a candidate biomarker for tumor incidence or prognosis requires further investigation. Detecting NDRG2 in combination with other molecules may contribute to the utility of NDRG2 in clinical settings. Wang et al. [15] detected NDRG2 and CD24 expression and found that the high NDRG2/low CD24 and low NDRG2/high CD24 combinations are independent prognostic indicators of lung adenocarcinoma that are also suitable for gallbladder carcinoma [55]. Jeschke et al. [85] identified the combination of HOXD1 and NDRG2 as the most sensitive (94%) and specific (90%) gene combination for detecting breast cancer. In HCC, the combination of low NDRG2/high phospho-STAT3 has prognostic value for adverse outcomes [74]. However, whether NDRG2 can be utilized as a biomarker for other types of cancer, with or without other molecules, requires additional research.

Epigenetic alterations play a key role in tumorigenesis, and inhibiting methylation or histone deacetylase processes with pharmaceutical interventions may have a benefit in cancer treatment. Trichostatin A is a histone deacetylase inhibitor, and NDRG2 expression is up-regulated by trichostatin A treatment via the inhibition of histone deacetylase [60, 72]. The methylation of NDRG2 is higher in primary gastric cancer specimens than in corresponding nonmalignant gastric tissues [60], and this pattern is also observed in OSCC [89]. Furthermore, upon treatment with a DNA demethylating agent, 5-aza-2′-deoxycytidine, NDRG2 expression is up-regulated in HGC27 cells, and MKN45 cell invasion is inhibited [60].

HT has been shown to alter the invasion capacity of cancer cells with few side effects [71]. Guo et al. [65] found that NDRG2 expression was induced by HT at 45°C. Moreover, the synergism between HT (43°C) and NDRG2 expression effectively reduces cytotoxicity and inhibits invasion compared with HT at 45°C. Thus, the combined application of constitutive NDRG2 expression with HT may yield an optimized therapeutic benefit. By increasing the phosphorylation of NDRG2, HT can also exert anti-tumor effects on MKN28 gastric cancer cells [71].

NDRG2 may decrease after antidepressant treatment and electroconvulsive therapy (ECT). Takahashi et al. [122] found that chronic treatment with imipramine, a tricyclic antidepressant, and sertraline, a selective serotonin reuptake inhibitor, reduced NDRG2 mRNA and protein expression in the rat frontal cortex. Moreover, repeated ECT significantly decreases NDRG2 expression in this region of the brain. These findings affirmed the important role of NDRG2 in the central nervous system and indicated that NDRG2 may be a candidate target of antidepressants and ECT, which suggests that NDRG2 can be induced by chronic stress. It is unknown whether cancer incidence and progression differs in people under chronic stress, which may contribute to a better understanding of the association between NDRG2 and cancer. The overexpression of NDRG2 in dorsal horn astrocytes contributes to their activation and plays a crucial role in diabetic mechanical allodynia. However, the intrathecal injection of RU486, a glucocorticoid receptor antagonist, reverses astrocyte reactivity and diabetic tactile allodynia by inhibiting NDRG2 overexpression [123]. Moreover, the use of glucocorticoids may induce NDRG2 expression [124], but whether this will contribute to the regulation of tumor development and progression remains unknown.

## CONCLUSIONS

NDRG2 expression is down-regulated in human cancer, and NDRG2 overexpression inhibits the proliferation, migration, metastasis and invasion of cancer cells (Table 1). There is a negative correlation between NDRG2 expression levels and the clinical and pathological status of human cancer (Figure 1). NDRG2 may be a tumor biomarker, and the combination of NDRG2 with other molecules, such as CD24 and HOXD1, may yield more specific or sensitive biomarkers. The anti-tumor effects of NDRG2 are exerted via various mechanisms and pathways (Figures 2 and 3), and NDRG2 expression levels are regulated by numerous factors and treatments, which may provide insight into methods for successfully treating cancer (Figure 1).
